# The concept of the sexual reproduction cycle and its evolutionary significance

**DOI:** 10.3389/fpls.2015.00011

**Published:** 2015-01-23

**Authors:** Shu-Nong Bai

**Affiliations:** State Key Laboratory of Protein & Plant Gene Research, Quantitative Biology Center, College of Life Science, Peking UniversityBeijing, China

**Keywords:** sexual reproduction cycle, meiosis, heterogametogenesis, fertilization, generation

## Abstract

The concept of a “sexual reproduction cycle (SRC)” was first proposed by Bai and Xu (2013) to describe the integration of meiosis, sex differentiation, and fertilization. This review discusses the evolutionary and scientific implications of considering these three events as part of a single process. Viewed in this way, the SRC is revealed to be a mechanism for efficiently increasing genetic variation, facilitating adaptation to environmental challenges. It also becomes clear that, in terms of cell proliferation, it is appropriate to contrast mitosis with the entire SRC, rather than with meiosis alone. Evolutionarily, it appears that the SRC was first established in unicellular eukaryotes and that all multicellular organisms evolved within that framework. This concept provides a new perspective into how sexual reproduction evolved, how generations should be defined, and how developmental processes of various multicellular organisms should properly be compared.

Sex is always a hot topic in human society and is an intensively investigated phenomenon in biology. Studies on sex determination in plants have focused on regulation of unisexual flower development (Ainsworth, 1999; Bai and Xu, 2013) since plant sex was defined based on unisexual flowers by Robbins and Pearson (1933), i.e., “a flower or plant is male if it bears only stamen and that is female if it bears only pistils.” However, plants with unisexual flowers account for only a small percentage of angiosperms, and angiosperms themselves are only some of the species in the plant kingdom. This raises the question of what sex is in angiosperms with perfect flowers (with both pistils and stamens) or in plants without flowers. Even further, do sex and sexual differentiation share any features in common in the plant and animal kingdoms?

To understand the regulatory mechanism of sex differentiation, following the concept originated from Robbins and Pearson (1933), we have investigated the regulation of unisexual flower development using cucumber for more than a decade. Cucumber is monoecious, and naturally bears both male and female flowers and, rarely, even hermaphrodite flowers, on the same plant. The ratio of male and female flowers can be affected by application of phytohormones such as ethylene (increasing the proportion of female flowers) or GA (increasing the proportion of male flowers). As hormones play key roles in mammalian sex expression, this phytohormonal regulation of the ratio of male and female flowers led to the use of cucumber a model system for research into plant sex determination starting in the 1960s (see Bai and Xu, 2013 and references there in). However, following the discovery that both male and female flowers contain initiated stamen and carpel primordia, we found that flowers become female because stamen development is inhibited early at stage 6, and that ethylene is involved in this inhibition (for detailed review, see Bai and Xu, 2013). These findings indicated that analysis of regulatory mechanisms in unisexual flowers will allow us to understand only how the inappropriate organs are inhibited, not how the appropriate organs are differentiated. We therefore referred to this situation as a “bird-nest puzzle,” meaning that it is not adequate to understand how a bird lays and hatches eggs through investigating how the nest was ruined (Bai and Xu, 2012).

Unisexual cucumber flowers are not the only example of this type of puzzle. All unisexual flowers in monoecious plants and many in dioecious plants for which the developmental mechanisms are known result from inhibition of one type of sexual organs (Bai and Xu, 2013; Akagi et al., 2014). To solve the “bird-nest puzzle,” we proposed a new way to define sex as a “heterogamete-centered dimorphic phenomenon,” sex differentiation as “the key divergence point(s) leading to the heterogamete differentiation” (Bai and Xu, 2013), which is in line with a definition previously suggested (Juraze and Banks, 1998), and generally applicable not only to all species in plant kingdom, but also in other kingdoms. We also hypothesized that before multicellular organisms emerged, a process called the sexual reproduction cycle (SRC) evolved based on the existing mitotic cell cycle in unicellular eukaryotes. This SRC starts from one diploid zygote, goes through meiosis, gametogenesis, and fertilization, and ends with two diploid zygotes. This review addresses the question of whether adopting the SRC concept can facilitate our understanding of sex.

## PREMISES FOR PROPOSAL OF THE SRC

We begin with the basic facts based on which the SRC was hypothesized.

Firstly, although DNA transmission in prokaryotes is sometimes referred to as “recombination” (Cavalier-Smith, 2002 and references therein), it is widely accepted that sex and sex differentiation are phenomena occurring in eukaryotes, which contain chromosomes, nuclei, cell skeletons, and the mitotic cell cycle, regardless of the obscurity of their evolutionary origins (Cavalier-Smith, 2002; Kirschner and Gerhart, 2005; Knoll, 2014). We would restrict our discussion in eukaryotes.

Secondly, meiosis is highly conserved in almost all known eukaryotes, including animals, plants, fungi, and protists (Logsdon, 2007; Schurko and Logsdon, 2008). It is also widely accepted that meiosis may originate from mitosis, probably via occasional mistakes in cohesin binding and/or digestion on chromosomes (Cavalier-Smith, 2002; Marston and Amon, 2004).

Thirdly, despite the extreme diversity of morphology and recognition mechanisms of gametes, cell fusion of two haploid gametes into a new diploid zygote is conserved in all eukaryotes.

Fourthly, while dimorphism of gametes is common in the organisms with which people are familiar, the smaller gametes being referred to as sperm and the bigger ones as eggs, there are also heterogametes that can be distinguished only at the molecular level, with different mating types such as *MAT*a/α in yeast and *MT*A/*MTD* in *Chlamydomonas* (Goodenough et al., 2007). Furthermore, heterogamy is not restricted to dimorphism, but rather can include multiple mating types, e.g., three in *Dictyostelium* and seven in *Tetrahymena* (Nanney and Caughey, 1953; Bloomfield et al., 2010; Cervantes et al., 2013). It is worth noting that such multiple mating types are mainly found in protists and fungi, but not in plant and animal kingdoms.

Fifthly, regardless of the presence or absence of germlines, e.g., in animals or plants, respectively, (Evens and Walbot, 2003), new generations in all multicellular organisms are generated through sexual reproduction consisting of the three key events: meiosis, sex differentiation, and fertilization. With this view, sexual reproduction is predicted to be more ancient than multicellular structures as sexual reproduction already existed in unicellular eukaryotic organisms.

There has been much debate about how sexual reproduction evolved (e.g., http://en.wikipedia.org/wiki/Sex; http://www.britannica.com/EBchecked/topic/536936/sex). It seems nearly impossible to obtain direct evidence regarding what happened during the period when sexual reproduction emerged, unless someday people can artificially “recapitulate” the evolutionary process. Nonetheless, we can try to explore the events involved in such evolution by analyzing the benefits for which the ancient evolutionary innovations could have been selected.

## MEIOSIS: A LUCKY MISTAKE?

The first indispensable event in sexual reproduction is considered to be meiosis. It is currently agreed that the most important benefit of meiosis is increasing genetic variation through recombination. However, by definition, meiosis is characterized by a reduction of chromosome numbers from diploid to haploid. How, then, did meiosis emerge and become selected?

It is known that haploid cells, like diploid cells, can undergo mitosis, such as in budding yeast. Considering the complexity of chromosome organization, it would be reasonable to speculate that the earliest eukaryotic cells were haploid. If that were the case, meiosis would be predicted to have evolved not only after the emergence of mitosis, but also after the emergence of diploid cells, which may have arisen from cell fusion or chromosome duplication in haploid cells.

While many organisms in the protist and fungus kingdoms live mainly in a haploid state (Campbell and Reece, 2005), almost all multicellular organisms in the animal and plant kingdoms use diploid cells as their building blocks. The prevalence of diploidy in the latter kingdoms suggests that diploidy must confer some advantages. If we naively believe that diploidy can doubly secure the genome stability of eukaryotic cells, then it follows that haploidy provides little leeway for mistakes. From this perspective, reduction of chromosome number would not be a good reason for meiosis to be selected. Instead, meiosis must occur and be selected for other reasons.

**FIGURE 1:**
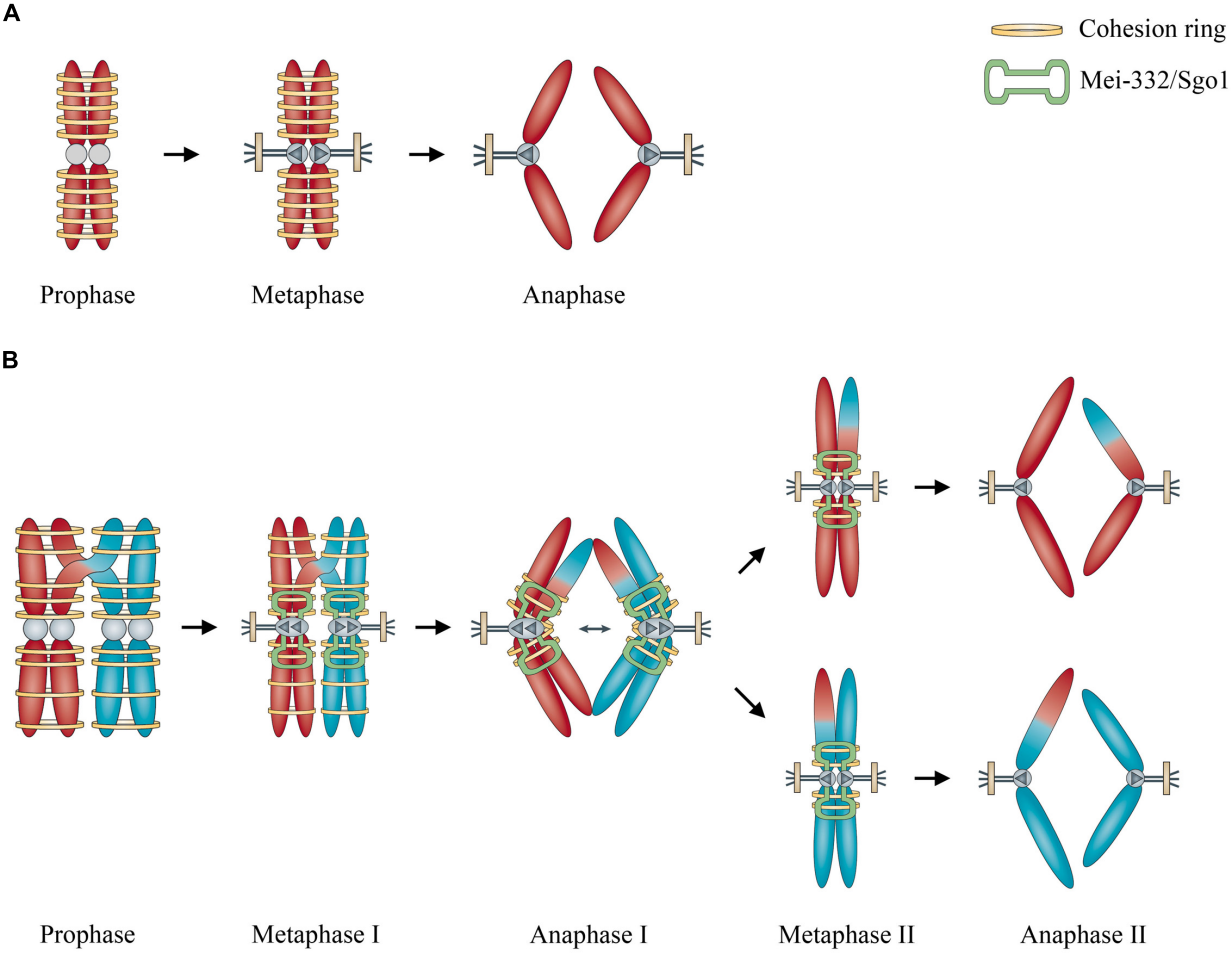
**Illustration of the role of cohesins in the origin of meiosis. (A)** Cohesins hold sister chromosome together during mitosis. **(B)** Cohesins mistakenly hold non-sister but homologous chromosomes together, enhanced by kinetochore-associated protein Mei-332/Sgo1 during meiosis. Modified from Marston and Amon (2004), by permission of Nature Publishing Group.

Based on Marston and Amon’s (2004) comparison of mitosis and meiosis, cohesins play important roles in both processes. In mitosis, cohesins like Scc3, Smc1, and Smc3 facilitate the cohesion of the two sister chromosomes, whereas in meiosis, the cohesins can hold together non-sister chromosomes from two different chromosomes (**Figure 1**). This might be analogous to playing ringtoss: the ring is thrown to capture a target, but sometimes the ring mistakenly captures something else together with the target. If cohesion is required for mitosis, mistaken association of non-sister but homologous chromosomes by cohesins may possibly occur like an off-target ring toss, and this may result in meiosis, facilitated by an ultimately meiosis-specific cohesin Rec8 and a kinetochore-associated protein MEI-S332/Sgo1 (Marston and Amon, 2004) and with abnormal degradation of cohesins afterward (Cavalier-Smith, 2002). Recently Ross et al. (2013) reported an evolutionary analysis on how a neogene acquired an essential function for chromosome segregation in *Drosophila melanogaster*, opening up a new perspective for investigating how the molecular mechanism of fundamental events like meiosis was evolved.

Why would a mistakenly occurring, “unnecessary” cell division be selected evolutionarily? Probably because of meiotic recombination. Although DNA transmission from cell to cell already existed in prokaryotes, meiotic recombination is considered to be the first efficient mechanism evolved for autonomously increasing genetic variation. This begs the question of why genetic variation would be so important for a cell that meiosis conferred an advantage during evolution. Regardless of how the first cell arose from an RNA world or pre-cellular biosystem, afterward the cells were relatively isolated from the environment from which they emerged. Although the advent of the cell granted the biosystem tremendous independence and the ability to proliferate itself through cell division, it created a problem of adapting to the unpredictable changes in its environment. Spontaneous DNA mutation is the original way to adapt, but with low efficiency. By contrast, meiotic recombination can generate numerous genetic variations more efficiently. Among the variations randomly generated during meiosis could be those that are adaptive to the prevailing environmental conditions and enable cell survival in a changed environment. Therefore, increasing genetic variation for adaptation might be a primary reason for meiosis to be selected. The stress-induced meiosis observed in protists is consistent with this speculation.

## DID FERTILIZATION EMERGE PRIOR TO MEIOSIS?

It is conventionally understood that the sexual reproduction starts from meiosis and ends at fertilization. However, as we speculated previously, it is likely that eukaryotes first emerged as haploid. If that is the case, cell fusion, rather than cell division, should be an ancient event in the emergence of diploid cells. Regardless of whether the first eukaryotic cell emerged when one prokaryotic organism engulfed another as Margulis and Sagan (1991) summarized and of how chromosome duplication originated, without diploid cells there would be no meiosis. Since fertilization is essentially a cell fusion process, we can predict that it is derived from the ancient cell fusion mechanism.

If indeed cell fusion arose prior to meiosis, once meiosis emerged in the resulting diploid cells, fertilization could be readily used as a mechanism to restore genome diploidy. Furthermore, if we think about the genetic variation randomly increased by meiosis, fertilization actually retains the variation derived from both cells that are the products of meiosis. In addition, considering the interaction between the meiotically produced cells and their environment, fertilization between the surviving haploid cells would actually execute a selective function in maintaining variations adaptive to that environment, as cells carrying non-adaptive variations would have not survived to participate in fertilization. Therefore, the advantages of fertilization include not only restoration of genome diploidy, but more significantly, double retention of the selected genetic variations generated through meiosis.

However, each meiotic cell would generate four types of resulting cells after random recombination. If those four cells randomly paired and fused, too much variation would rapidly diversify the characteristic genome structure of the species along with the increase of round from one zygote to zygotes of next generations. This does not even take into account the genetic complexity from a population perspective, in which the meiotically produced cells could pair and fuse with those arising from other meiotic cells. Is there any way to solve that problem?

## A BENEFIT OF HETEROGAMY: LABELING MEIOTICALLY PRODUCED CELLS TO HARNESS VARIATION WHILE ENHANCING HETEROGENEITY

Heterogametes in animals and plants generally display morphological differences, i.e., small sperm and large egg cells in comparison with their progenitor meiotic cells. However, as mentioned above, heterogamy in unicellular eukaryotic organisms is frequently determined at the molecular level by a single genetic locus, and can include more than two mating types. If we use the assumption that unicellular eukaryotes evolved prior to multicellular organisms, we may infer that the morphological and/or physiological differentiation of heterogametes in multicellular organisms should be considered elaborations of the very simple differences, such as at a single genetic locus, observed in gametes of unicellular eukaryotes. This inference is consistent with the recent finding that in *Volvox*, expansion of a mating locus causes heterogametes to change from being equal in size to being dimorphic (Ferris et al., 2010).

What is the advantage of heterogamy that enables the mating loci to be selected among the enormous genetic variation? If we remember the problems mentioned above regarding random pairing and fusion of meiotically produced cells in fertilization, we can speculate that heterogamy would significantly restrict diversity. With heterogamy, meiotically produced cells are classified into different groups that prevent pairing and fusion of those among the same group. In other words, heterogamy is essentially a mechanism of labeling meiotically produced cells to prevent “self-mating,” therefore “harnessing,” while enhancing, heterogeneity generated from meiosis and fertilization. As it allows pairing and fusion only between haploid cells from different groups, this harnessing mechanism creates a relatively stable interval during which the adaptive cells can be selected.

If the essential function of heterogamy is the labeling of meiotically produced cells and thereby harnessing variation while enhancing heterogeneity, there should be a multitude of ways to achieve this. Genetic loci for mating types probably represent the most ancient and simple way, but there could be many other modifications to enhance the differentiation for higher efficiency.

**FIGURE 2:**
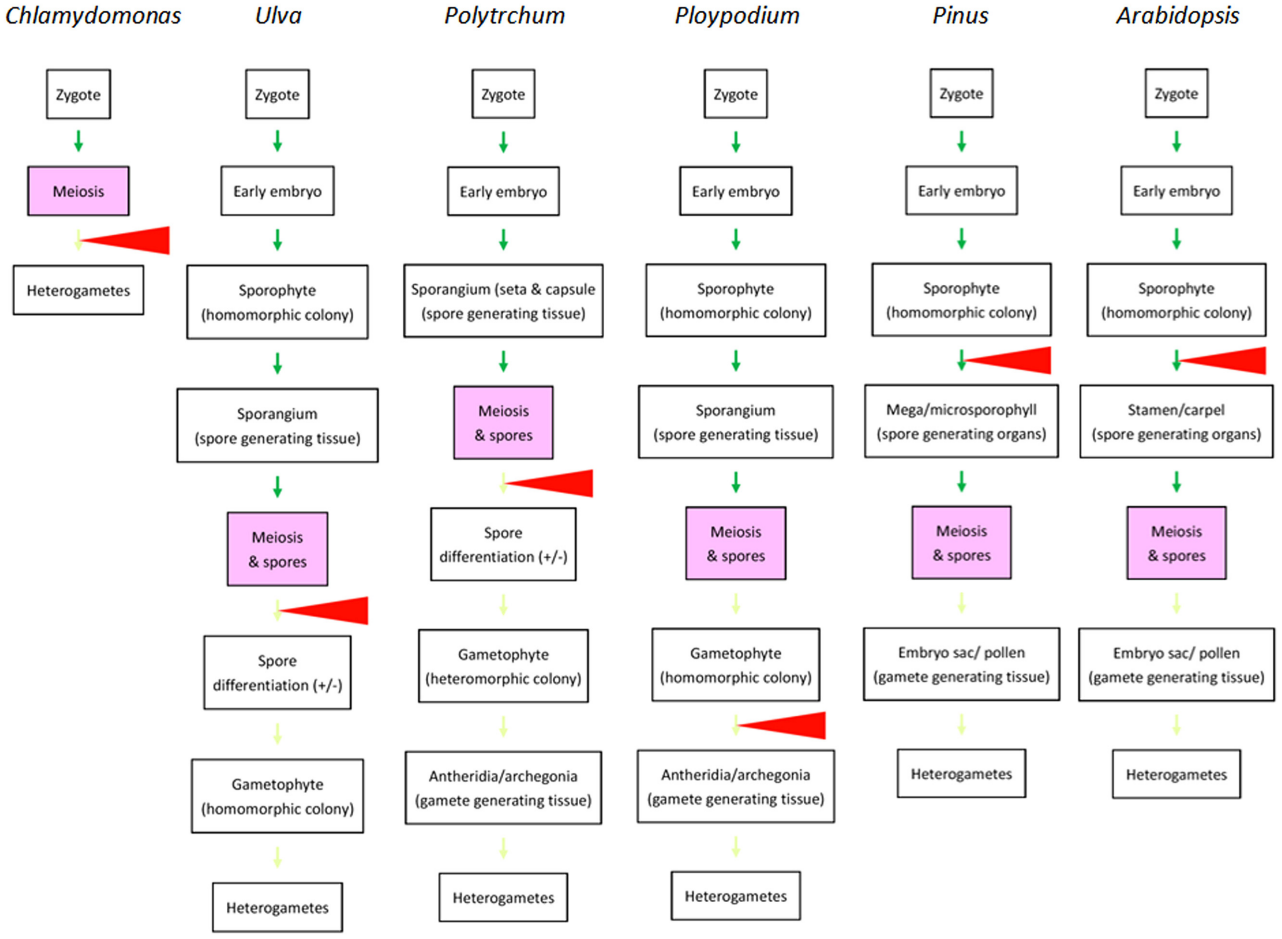
**Comparison of life cycles of various autotrophic organisms emphasizing the divergence points resulting in dimorphic structures related to heterogametogenesis.** From left to right: life cycles of selected species representing unicellular green algae (*Chlamydomonas*), multicellular green algae (*Ulva*), mosses (*Polytrichum*), ferns *Ploypodium*), gymnosperms (*Pinus*), and angiosperms (*Arabidopsis*) are briefly outlined. Green arrows indicate morphological transitions in sporophyte generations. Light green arrows indicate morphological transitions in gametophyte generations. Red triangles indicate the major divergence points leading to dimorphic development for heterogametogenesis. The major divergence points are shifted from post-meiosis in green algae, mosses, and ferns (in some species like *Ploypodium*) to before meiosis in gymnosperms and angiosperms. Reprinted from Bai and Xu (2013), by permission of Elsevier.

In majority of animals familiar to human experience, heterogametes are differentiated from germlines that migrate into and complete the differentiation in dimorphic gonads during embryogenesis. Heterogamy is determined mainly in gonad differentiation prior to germ cells undergoing meiosis. One may therefore believe that sex determination or differentiation is a precondition of the occurrence of meiosis. The situation appears similar in plants if we examine only angiosperms. However, if we take ferns and mosses in consideration, it is easily found that dimorphism of multicellular structures is not necessarily required for meiosis and that heterogamy in multicellular organisms can be achieved after meiosis (**Figure 2**), similar to what takes place in *Chlamydomonas* and *Volvox* (Goodenough et al., 2007; Ferris et al., 2010). If we compare the divergence points in green algae and the four groups of land plants, we see a trend in which the divergence point(s) that leads to the heterogamete differentiation shifted from gametophytes after meiosis to sporophytes before meiosis in green algae and angiosperms, respectively. Little is known regarding how this shift evolved. However, efficiency in gamete distribution and meeting might contribute to the shift: in *Chlamydomonas*, the two types of gametes differentiate in water and their pairing and fusion occurs randomly. In mosses and ferns, sperm cells are shed into water as well and swim to archegonia and eggs with water as the medium. In gymnosperms and angiosperms, in which the divergence points are shifted to sporophytes, the delivery of sperm is no longer restricted to water. This may allow these two groups of plants to increase their spatial distribution.

Similarly, if we examine mechanisms of animal sex differentiation with a broader view, there are also diversifications worth noting: although gametogenesis is carried out in the germlines, sex differentiation mainly occurs at the gonads. While mammalian gonad differentiation from bipotential to unisexual is triggered by sex-determining genes, a similar gonad differentiation is induced by environmental temperature in some reptiles (Ramsey and Crews, 2009). This implies that over the course of evolution there might be a trend in which determination of heterogametogenesis shifted from germ cells in *cis* to somatic gonads in *trans*, and further that the trigger(s) for gonad differentiation shifted from environmental signals to genetic factors encoded in chromosomes, and even further that the chromosomes bearing genetic factors determining sex evolved into sex chromosomes, as suggested by Charlesworth et al. (2005).

If the above speculation is accurate, sex differentiation indeed can be considered essentially a labeling mechanism for heterogamy, regardless of how diversified in form and complicated in regulation, in a wide spectrum of organisms from unicellular eukaryotes to multicellular animals, plants, and fungi.

## SEQUENTIAL DIFFERENTIATION OR INTEGRATION OF INDEPENDENTLY EVOLVED EVENTS?

In animals, meiosis and gametogenesis occur sequentially in germlines and sex differentiation occurs in somatic gonads into which the germline migrated. In plants, meiosis and gametogenesis occur separately from somatic cells of the sporophyte and gametophyte, while sex differentiation could occur either in sporophyte or gametophyte. How were meiosis, gametogenesis, and sex differentiation originally integrated? Considering that all three processes exist in protists, one possible scenario is that each evolved independently, and they were integrated together as a coordinated process by chance and thereafter genetically fixed as a program in protists. This scenario is possible because all three processes, meiosis, heterogametogenesis (including sex differentiation and gametogenesis), and fertilization, occur at the cellular level. Protists are unicellular eukaryotes and live in a population. These two characteristics provide the required conditions for SRC emergence: on one hand each cell can behave independently for emergence of meiosis and gametogenesis, and on the other, all cells live together closely enough to make both cell fusion and cell–cell recognition possible. Integration of the three events would have brought all of their selective advantages together and such integration, now referred as SRC, would be therefore selected during evolution.

## MODIFIED CELL DIVISION: ORIGIN OF GENERATIONS

**FIGURE 3:**
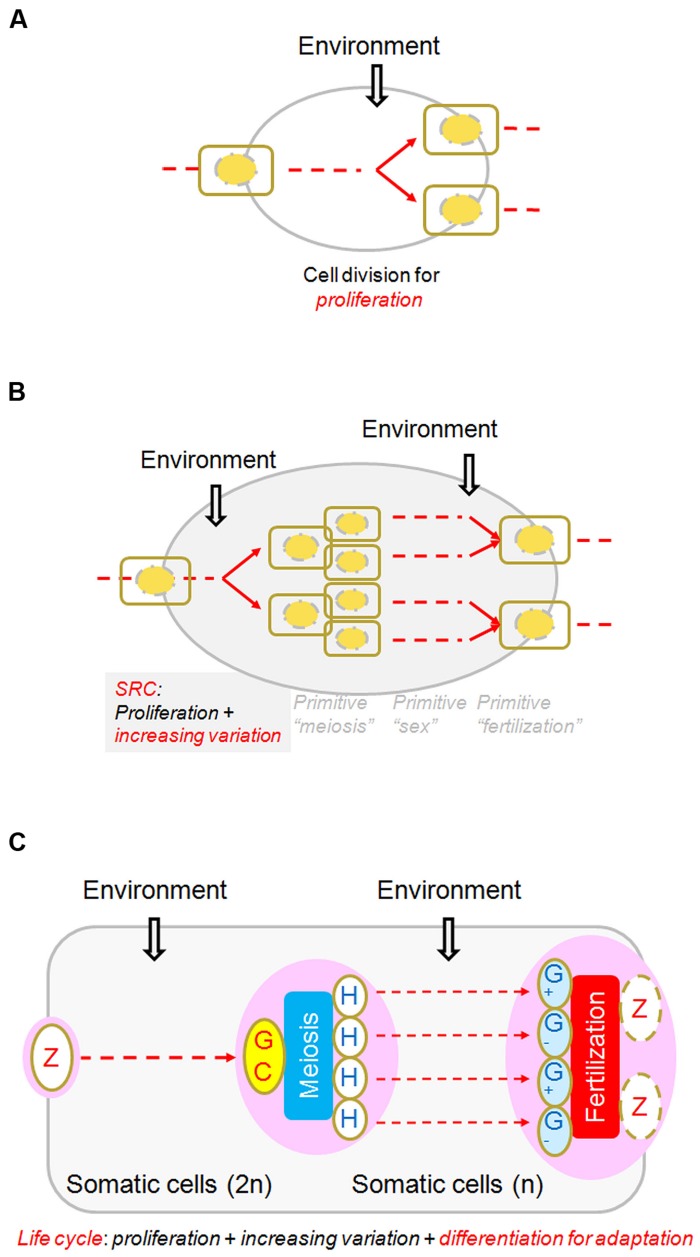
**Diagram of the sexual reproduction cycle (SRC). (A)** A regular cell cycle for proliferation, through which one cell becomes two, and environmental conditions trigger or affect the cycle at various points in the process. **(B)** A “SRC.” Three hypothetically random and independent events, “primitive meiosis,” “primitive fertilization,” and “primitive sex,” occasionally integrated and were selected for advantages in adaptation. The net result of the SRC is that one cell becomes two, just as in the regular cell cycle, regardless of how these events evolved and were integrated (of which little is known). **(C)** Core processes of the life cycles of multicellular organisms. From the perspective of the SRC, it is clear that all multicellular structures arose in the interval phases of the SRC through the regular cell cycle and cellular differentiation, whether diploid (in almost all organisms) or haploid (mainly in plants and fungi). Modified from Bai and Xu (2013), by permission of Elsevier.

In nearly all biology textbooks, meiosis is introduced in comparison with mitosis, whereas fertilization and sex determination or differentiation are introduced elsewhere. However, if we view meiosis and fertilization together, we find that one cell becomes four (except in some particular cases, such as angiosperms and mammalian, only one female meiotically produced cell remaining alive to differentiate into female gamete) through meiosis and two cells become one through fertilization. Thus, the net result of the entire SRC is that one cell becomes two, just like one round of mitosis. The fundamental difference between the SRC and the mitotic cell cycle is that the genetic compositions of the two cells resulting from the SRC are different from that of the progenitor while the products of the mitotic cell cycle remain similar to that of their progenitor (**Figure 3**). It needs to be emphasized that what can appropriately be compared with the mitotic cell cycle is not meiosis alone, but rather the entire process of SRC, including meiosis, heterogametogenesis, and fertilization.

Cell division occurred well before eukaryotes evolved. Despite the difference in complexity between mitotic cell division in eukaryotes and cell fission in prokaryotes, the two processes are similar in that the two resulting cells retain the same genome structure as the starting cell. By contrast, the genome structures of the two cells resulting from the SRC are no longer the same as that of the original cell, as described above. They are genetically a new “generation.” From this perspective, although the terms “mother cells” and “daughter cells” are often used in describing the beginning and resulting cells in mitosis, these cells do not truly represent two generations. They are actually clones, the same as in the cell division observed in *Escherichia coli* leading to a proliferation of the same generation. Only through the SRC is a new generation created.

According to Chen et al. (2013), genetic variations are generated in several ways, such as mutation and new gene origination, in addition to meiotic recombination. However, only variations retained through the SRC can be maintained from one generation to the next, rather than being diluted and ultimately disappearing through continuous cell divisions. In that sense, mainly because it was integrated as part of the SRC, meiotic recombination took a prominent position among the various ways of creating genetic variations.

## THE SRC: AN APPENDIX OF A MULTICELLULAR ORGANISM OR A FRAMEWORK ONTO WHICH MULTICELLULAR STRUCTURES ARE INTERPOLATED?

When animal development is discussed, an organism and embryogenesis takes center stage. Germline initiation is an appendant event during embryogenesis, while meiosis and gametogenesis are only two events of germline differentiation. A similar situation occurs in our understanding of plant development. The focus is mainly on the morphogenesis of multicellular structures. However, if we take the standpoint that the SRC emerged in protists, an obvious inference is that multicellular structures all emerged within the framework of the SRC. Is this possible?

If we accept the argument that cell growth and division are coupled with optimal cell volume (Buchanan et al., 2000), and the inference that meiosis is a stress-induced specific cell division for adapting to unpredicted environmental challenges, we can imagine that under optimal environmental conditions, a cell would keep dividing in order to maintain optimal cell volume. That means that between a zygote and a meiotic cell, and between meiotically produced cells and gametes, there would be two intervals during the process completing the SRC (**Figure 3C**). In unicellular protists, cells in these two intervals would be mainly freely living in a population. However, under certain conditions, for example nutritional shortages, the free-living cells might be aggregated or organized, such as in *Dictyostelium* (Dormann et al., 2002) and *Volvox* (Kirk, 2005). What would result if the conditional aggregations or organizations were genetically fixed? Multicellular organisms! If this speculation is valid, what is the relationship between the core cells involved in the SRC, such as the zygote, meiotic cells and gametes, and those involved in multicellular structure morphogenesis? A reasonable answer is that the SRC serves as a framework or backbone, within or on which multicellular structures consisting of “somatic cells” could interpolated into the two intervals when the environment was optimal. Different from simply maintaining cell volume through cell division, organized multicellular structures ultimately facilitate energy acquisition and environmental adaptation. All elaborated sex differentiation and mating behaviors can be viewed as nothing more than modifications of multicellular structures evolved afterward to facilitate meeting, recognition, and proper fusion of the two gametes.

**FIGURE 4:**
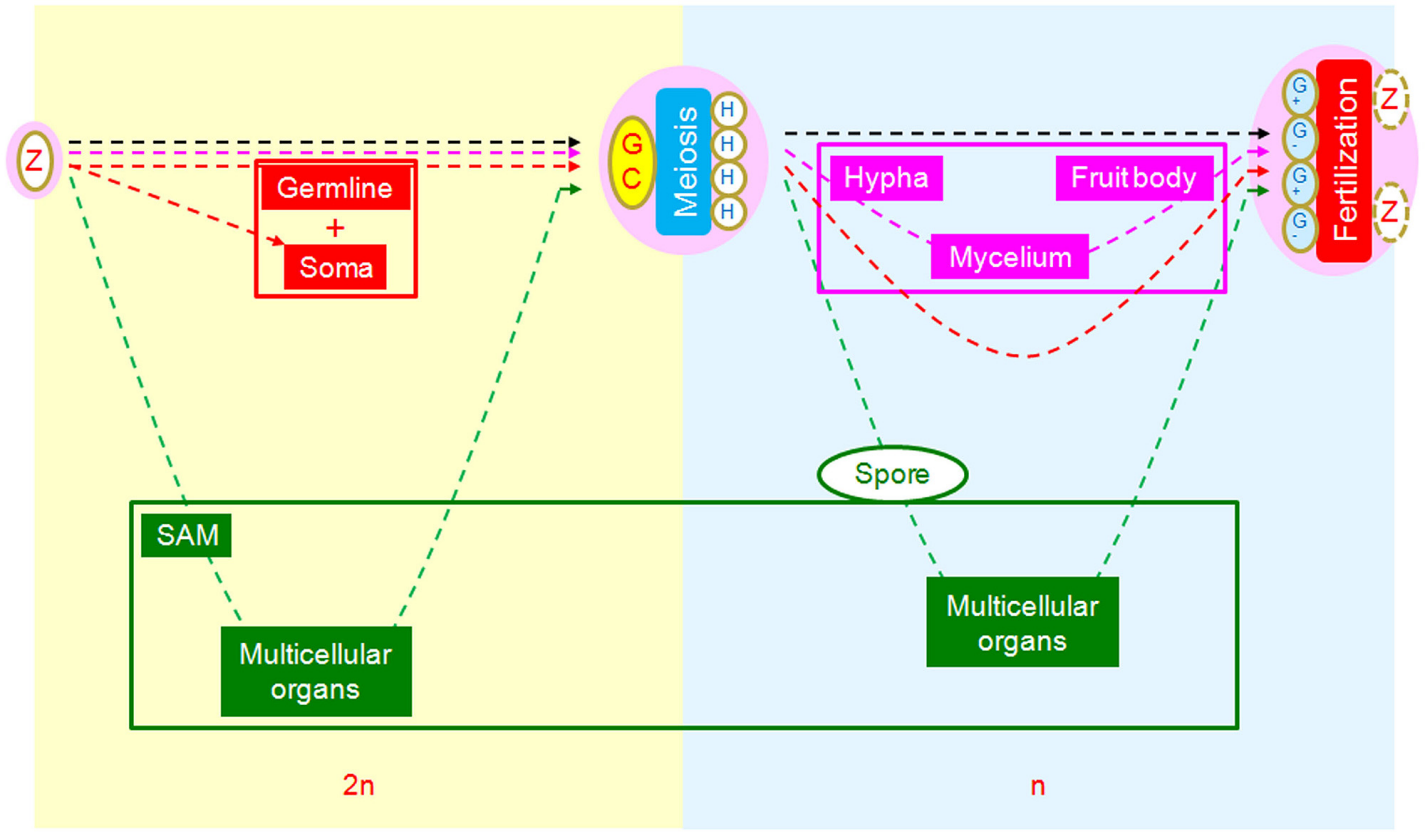
**Comparison of morphogenetic strategies of animals, fungi, and plants in the framework of SRC.** Yellowish background indicates diploid phase and bluish haploid phase. In the intervals between zygote and germ cells, multicellular structures of animals (red) and plants (green) are interpolated, while there are none in fungi (pink). In the intervals between meiotically produced cells and gametogenic cells, multicellular structures are interpolated for fungi and plants but not animals.

From this perspective, we can also see a relatively novel scenario in which to compare the core developmental processes of animals, plants, and fungi using the SRC as a common reference framework (**Figure 4**). From this comparison, we observe three different strategies of morphogenesis. In animals, the multicellular structures (soma) are interpolated at the first interval between zygote and meiotic cells as rest of embryos in addition to germlines. In fungi, the multicellular structures are interpolated at the second interval between meiotically produced cells and gametogenic cells, with unknown mechanisms of cell aggregation (Meskauskas et al., 2004; Lee et al., 2010). In plants, multicellular structures are interpolated at both intervals, with an additive strategy (Bai and Xu, 2013).

## CONCLUSION: THE SRC AS A KEY EVOLUTIONARY INNOVATION

Taking the above analysis together, several speculations can be made: first, meiosis is the most effective mechanism evolved to autonomously increasing genetic variation; second, the SRC is the first known genetic program of cell differentiation and coordination, integrated with the three independently evolved events, i.e., meiosis, sex differentiation, and fertilization, among cell populations; third, with the SRC, eukaryotes were first equipped with an internal mechanism to adapt effectively to unpredictable environmental challenges. From this perspective, the SRC deserves to be recognized as a key evolutionary innovation. This innovation first arose in unicellular eukaryotes, and then, because of its advantages in adaptation to environmental challenges, was adopted by subsequent multicellular organisms as a conserved program. Only based on the SRC can generations be properly defined, thereby allowing genetic analysis of biological processes. In addition, SRC establishes a reasonable framework in which to understand how multicellular organisms evolved in various kingdoms, including animals, plants and fungi.

I used to be highly curious about why the developmental processes of multicellular organisms are unidirectional, i.e., from zygotes to gametes. After I became aware of the existence of the SRC, this puzzle seemed easy enough to be solved because the SRC is a program that originally emerged and was selected as a response to environmental stresses. As environmental stresses occur ultimately independent of organisms and irreversible, the SRC process is irreversible and therefore unidirectional. Although the three core events in the SRC of many multicellular organisms have been genetically encoded during evolution and may not directly respond to environmental stresses, the unidirectionality was inherited from their unicellular eukaryotic ancestors.

Another issue confusing for a long time is how to understand the role of “asexual reproduction” in biological processes, such as speciation (Coney and Orr, 2004). With the concept of the SRC, it is clear that only the organism multiplication that is coupled with the SRC should be regarded as “reproduction,” as new generations are produced. Multiplication of organisms not coupled with SRC should not be regarded as “reproduction,” as no new generations are produced. Instead, the products of the latter are properly referred to as clones, equivalent to mitotic cell division, regardless of how complicated and similar the process is in comparison to the process starting from a zygote. In that sense, the past comparisons of “sexual reproduction” vs. “asexual reproduction” should more correctly be of “reproduction” vs. “proliferation.” The former refers to a multiplication of organisms based on the SRC, from one generation to the next in a lineage, while the latter refers to a multiplication of organisms remaining within the same generation.

One more issue worth noting here is that the SRC is essentially a mechanism allowing diploid cells to adapt to unpredictable environmental challenges. If an organism adapts to its environment in the haploid form, there would be no selective pressure for it to adopt SRC. This can explain why there are numerous examples of meiosis and fertilization occurring only rarely in many haploid-dominant protists (Logsdon, 2007), and no stable SRC identified in many haploid-dominant fungi (Heitman et al., 2013).

In his book “Primitive Land Plants” first published in 1935, Bower (1935) had advised that in understanding plant morphology, we should adopt “upward comparative analysis,” i.e., use forms of organisms that emerged early as a reference framework, and treat the new traits in organisms that emerged later as modifications upon the earlier forms. Similar principles can also be applied to understanding sex and sexual reproduction.

Lastly, I would like to emphasize that without the investigation of unisexual flowers, which brought about the “bird-nest puzzle,” and subsequent historical retrospection of the definition of sex in plants, I would not have this chance to think about the essential role of sex in living systems. In addition, without analysis of highly diversified sex differentiation, i.e., divergence points leading to heterogametes in various plant groups, it would be impossible to disconnect the sex or sex differentiation from meiosis, as in all animal systems, sex differentiation always occurs prior to meiosis, and therefore meiosis appears undeniably linked to sex differentiation. In many aspects, our views of biological processes in plants have been borrowed from those developed in animal research, into which vastly more resources have been invested since we, as humans, belong to the animal kingdom. Sometimes, however, plants provide an invaluable alternative due to their different morphogenetic strategies. Ultimately, how we look at biological events depends on what questions we address. If we want to understand fundamental principles of living systems, we need to properly compare animals and plants at least, as each can be used as a control for the other. Alternatively, if one only wants to know the mechanism of animal disease or crop yield for instance, that comparison might be not necessary. For questions about sex *per se*, proper comparisons among animals and plants, plus fungi, and more importantly protists, seem indispensable!

## Conflict of Interest Statement

The author declares that the research was conducted in the absence of any commercial or financial relationships that could be construed as a potential conflict of interest.
